# Technological Assessment and Predictive Modeling of Probiotic Lactose-Free Fermented Milk with *Lacticaseibacillus paracasei* GV17

**DOI:** 10.3390/foods14071176

**Published:** 2025-03-27

**Authors:** Taynan Jonatha Neves Costa, Isabella Maciel Costa, Larissa Mirelle Mendes Magalhães, Marcelo Resende de Souza, Gabriel Augusto Marques Rossi, Bruna Maria Salotti-Souza, Camila Argenta Fante

**Affiliations:** 1 Department of Food, Faculty of Pharmacy, Federal University of Minas Gerais, Belo Horizonte 31270-901, Brazil; taynanjncosta@gmail.com (T.J.N.C.); larissamirellemm@gmail.com (L.M.M.M.); camila.fante@gmail.com (C.A.F.); 2 Department of Technology and Inspection of Animal Products, Veterinary School, Federal University of Minas Gerais, Belo Horizonte 31270-901, Brazil; bellamaciel@hotmail.com (I.M.C.); marceloresende51@gmail.com (M.R.d.S.); 3 Department of Veterinary Medicine, University Vila Velha (UVV), Vila Velha 29102-920, Brazil; gabriel.rossi@uvv.br

**Keywords:** lactic acid bacteria, growth kinetics, mathematical modeling, probiotics

## Abstract

This study investigated *Lacticaseibacillus paracasei* GV17, a potentially probiotic strain, in association with the commercial culture *Streptococcus thermophilus* STI-12, in lactose-free fermented milk. Predictive modeling was used to estimate growth parameters and microbial viability and the technological characteristics of the fermented milk during storage. The initial concentrations of the strains were 9.80 log CFU/mL for *Lc. paracasei* GV17 and 9.50 log CFU/mL for *S. thermophilus* STI-12. After eight hours, the pH reached 4.6, and the concentrations of GV17 and STI-12 were 10.90 log CFU/mL and 11.20 log CFU/mL, respectively. The Baranyi model was fitted to the growth data, with correlation coefficients of 0.760 for *Lc. paracasei* GV17 and 0.852 for *St. thermophilus* STI-12. The maximum specific growth rates were 0.912 log CFU/h for GV17 and 0.882 log CFU/h for STI-12. Regarding technological characteristics, syneresis decreased by 8.90% after 28 days, indicating greater structural stability, while water retention capacity remained constant. The viability of LAB remained above 10.00 log CFU/mL. *Lc. paracasei* GV17 showed great potential for use in functional products, prompting further research.

## 1. Introduction

The extensive application of lactic acid bacteria (LAB) in the food industry goes beyond their food preservation characteristics to include the diverse flavors and aromas generated during the fermentation process. There is no doubt that LAB are a valuable economic asset for the food industry, especially in the dairy sector [1].

Understanding strategies to prevent food spoilage and contamination by foodborne pathogens is a widely debated issue in the food industry [2]. Given their metabolic capabilities, LAB species are regarded as promising candidates to suppress the growth of foodborne pathogens and prolong product shelf life in food processing [3].

LAB are known for adapting to the dairy food matrix and contributing to the fermentation process of dairy products. The combination of commercial LAB cultures with probiotics in milk fermentation gives the product functional properties [4]. A rising concern in the food industry is the expanding lactose-free market. It is estimated that 75% of the global population lacks sufficient β-galactosidase, the enzyme necessary for lactose hydrolysis [5].

Fermented milk stands out among dairy products for their sensory appeal. While fermentation leads to partial lactose hydrolysis, a substantial portion of the disaccharide remains intact, requiring additional hydrolysis through the use of exogenous lactase [6].

The study of mathematical models to calculate the growth and survival kinetics of bacteria has been widely used. The application of these models can predict the microbial behavior of LAB. It is important to note that fermentation is one of the key methods for producing compounds of probiotic interest [7].

Predictive microbiology scrutinizes variables that delineate the behavior of microorganisms within food matrices, offering a swift and dependable framework for comprehending microbial proliferation, inactivation, and survival under distinct conditions [8]. The mathematical model developed by Baranyi and Roberts is categorized as an empirical, kinetic, and primary model, which enjoys extensive utilization and application in the realm of predictive microbiology [9].

In this context, this work aimed to estimate the growth parameters of *Lacticaseibacillus paracasei* GV17, associated with the commercial coculture *Streptococcus thermophilus* STI-12, using predictive modeling. Additionally, it sought to evaluate the microbial viability of lactic acid bacteria and the technological characteristics of lactose-free fermented milk during the storage period.

## 2. Materials and Methods

### 2.1. Lactic Acid Bacteria

The potentially probiotic strain *Lacticaseibacillus paracasei* GV17 [10], from the Culture Collection of the Department of Technology and Inspection of Products of Animal Origin at the Federal University of Minas Gerais, was used (DTIPOA/UFMG, Belo Horizonte, MG, Brazil). The LAB was previously isolated from the processing of Minas artisanal cheese from the Campo das Vertentes region in Minas Gerais and identified using the MALDI-TOF spectrometry technique. For MALDI-TOF mass spectrometry, the Microflex™ system (Bruker Daltonics, Brémen, Germany) and its database were used. A fresh bacterial colony was transferred from Petri dishes to a stainless-steel target plate; this was followed by the addition of 1 μL of formic acid (70%) and 1 μL of α-cyano-4-hydroxycinnamic acid. The plate was then inserted into the equipment, and the mass spectrum generated was compared with the database. Identification criteria were the ones recommended by the manufacturer, i.e., scores ≥ 2.000 = species-level; ≥1.700 and <2.000 = genus-level, and <1.700 = unreliable identification [11]. *Lacticaseibacillus paracasei* GV17 achieved a score of ≥2000 for species-level identification.

GV17’s probiotic potential has been previously evaluated in vitro [10]. Antimicrobial susceptibility testing revealed that GV17 is sensitive to clindamycin, chloramphenicol, and erytromycin. Additionally, it demonstrated a hydrophobicity of 32.88% and autoaggregation rates exceeding 41% at 4 °C, 37 °C, and 42 °C. Furthermore, GV17 resisted simulated gastrointestinal tract (GIT) conditions with counts higher than 8.00 log CFU/mL after the enteric phase.

*Lc. paracasei* GV17 was cultivated in Man–Rogosa–Sharpe (MRS) broth (Difco, Becton Dickinson Co., Sparks, MD, USA) and subsequently preserved at −80 °C in conjunction with 20% (*v*/*v*) sterile glycerol employed as a cryoprotective agent. For the purpose of reactivation, the isolated lactic acid bacterium was introduced (2%, *v*/*v*) into 10 mL of MRS broth and incubated under aerobic conditions for a duration of 24 to 48 h at a temperature of 37 °C [12].

### 2.2. Culture Conditions

#### 2.2.1. Viability of LAB in Glucose and Lactose Medium

For the fermentation adaptation experiments, lactose, glucose, and 50% lactose and 50% glucose (glucose–lactose) media were used, prepared as Yamamoto et al. [13]. The lactose medium (Lac) contained only lactose as a carbon source and was prepared by sterilizing a milk broth containing 10% (*w*/*w*) skimmed milk powder (Molico^®^, Nestlé, Araçatuba, São Paulo, Brazil) by heating it at 95 °C for 2 min. The glucose medium (Glu) was prepared with 10% (*w*/*w*) lactose-free milk powder (Nolac^®^, Itambé, Belo Horizonte, Minas Gerais, Brazil) and heated at 95 °C for 2 min. In the glucose medium, lactose was completely hydrolyzed into glucose and galactose.

For the monoculture of GV17, the preculture was inoculated at 0.2% (*v*/*v*) in lactose, glucose, and glucose–lactose (Glu:Lac) media. The same procedure was repeated for the monoculture of STI-12. For the coculture, the precultures of GV17 and STI-12 were inoculated at 0.1% (*v*/*v*) of each LAB culture into Lac, Glu, and Glu:Lac media. All cultures were fermented at 40 °C under aerobic conditions until a pH of 4.6 was reached.

#### 2.2.2. Determination of pH

The pH during fermentation in Lac, Glu, and Glu:Lac media was measured using a calibrated pH meter (Gehaka PG1800 digital pH meter, São Paulo, Brazil) at one-hour intervals. The time required for the pH to reach 4.6 was defined as the fermentation time.

#### 2.2.3. Assessment of the LAB Population

After the media reached a pH of 4.6, the LAB population was counted. The strains were cultured on MRS agar for the *Lc. paracasei* GV17 count and on M17 agar for the *S. thermophilus* STI-12 count. The plates were incubated aerobically for 48 h at 37 °C. Viability was expressed as log CFU/mL.

### 2.3. Kinetic Profiles of LAB Strains in Fermented Milk

The *Lc. paracasei* GV17 strain was activated and collected by centrifugation (6000× *g* for 6 min at 4 °C), followed by two washes and dilution in 0.85% sterile saline solution, and centrifuged again under the same conditions. The LAB cells were then resuspended in 10 mL of sterile reconstituted lactose-free whole milk powder (Nolac^®^, Itambé, Belo Horizonte, Minas Gerais, Brazil). *Streptococcus thermophilus* STI-12 (Chr-Hansen^®^, Cadorago, Como, Italy) (0.015 g) was suspended according to the manufacturer’s instructions in 50 mL of lactose-free whole milk powder and activated at 42 °C for 30 min.

#### 2.3.1. Milk Fermentation

The lactose-free whole milk powder was reconstituted in water to achieve 12% total solids (*w*/*v*) and added with 7% sugar (Guarani^®^, Olímpia, São Paulo, Brazil). The milk was heated to 90 °C for 10 min using a Thermomix (Cloyes-sur-le-Loir, France) and then cooled to 42 °C in an ice-water bath, and 4% inoculum (8 log CFU/mL) was added, with GV17 and STI-12 being added in a 1:1 ratio. The heat-treated milk contained *Lc. paracasei* GV17 cocultured with the commercial strain *S. thermophilus* STI-12.

The milk, after adding the cultures, was dispensed into sterile 50 mL bottles and fermented at 42 °C until a pH of 4.6 was reached. The selected fermentation temperature was found to be adequate for the strains, based on preliminary tests conducted on the autochthonous (GV17) and commercial (STI-12) cultures. After fermentation, the milk was cooled to 15 °C, and the gels were broken. The product was then stored at 4 °C for 28 days to evaluate LAB viability, syneresis, and water retention capacity.

#### 2.3.2. Assessment of the LAB Population in Fermented Milk

To count the LAB population during fermentation, samples were collected at regular intervals of 30 min until a pH of 4.6 was reached. LAB strains were grown using pour plating on MRS agar for counting *Lc. paracasei* GV17 and on M17 agar for counting *S. thermophilus* STI-12. The plates were incubated aerobically for 48 h at 37 °C. Viability was expressed as log CFU/mL of fermented milk.

#### 2.3.3. Determination of Titratable Acidity and pH Throughout Shelf Life

On the 1st, 14th, and 28th days after milk fermentation, titratable acidity and pH were evaluated. A 10 mL sample was taken for titratable acidity evaluation, and 10 mL of carbon dioxide-free water was added and mixed using a glass rod. Then, five drops of phenolphthalein solution were added, and titration was performed using 0.1 M sodium hydroxide solution until a pink coloration appeared [14]. pH determination was performed using a calibrated pH meter (Gehaka PG1800 digital pH meter, São Paulo, Brazil).

#### 2.3.4. Estimation of LAB Multiplication Parameters During Fermentation

To estimate the multiplication parameters, Equations (1) and (2), proposed by Baranyi and Roberts [9], were used. The lag time (λ, h), multiplication rate (μ_max_, h^−1^), and maximum population were estimated (log CFU/mL).

The Baranyi model incorporates a latency term based on biological processes, accounting for the adaptation of bacteria to the new environment. In other words, it represents the behavior of bacterial cells before the onset of the exponential phase. Additionally, it describes the growth of LAB under various environmental conditions, as it more accurately captures the adaptation, exponential growth, and stationary phases [15].

(1) $$y\left(t\right)=y_{0}+\mu_{max}A\left(t\right)-In\left(1+\frac{exp\;\left({µ}_{max}A\left(t\right)\right)-1\;}{exp\;\left(y_{max}-y_{0}\right)}\right)$$

(2) $$A\left(t\right)=t+\frac{1}{\mu_{max}}\ln\left(\frac{e^{\left(-\mu_{max}t\right)}+q_{0}}{1+q_{0}}\right)$$

In this context, y(t) represents the cell concentration (log CFU/mL) at time t (days), while y_max_ (k) is the maximum cell concentration (log CFU/mL). The term y_0_ refers to the initial cell concentration (log CFU/mL), and µ_max_ denotes the maximal specific growth rate (CFU/mL/day). q_0_ measures the physiological state of the cells, and mm is a parameter that describes the curvature after the exponential growth phase. Additionally, A(t) is a function that accounts for the physiological state of the cells, incorporating the effects of various factors on microbial metabolism.

Parameters were estimated using Microsoft Excel Dmfit version 3.5 (ComBase, Dresden, Germany, https://www.combase.cc/ (accessed on 25 November 2024). With the Baranyi model formula [9], were used to obtain the maximum growth rate (µ_max_) and R^2^ (global measure of prediction fidelity).

#### 2.3.5. Determination of Syneresis Throughout Shelf Life

Syneresis was assessed in triplicate on the 1st, 14th, and 28th days after milk fermentation. The syneresis estimation was carried out using the method outlined by Kieserling et al. [16]. Approximately 15 g of fermented milk samples were weighed and placed in a 15 mL Falcon tube. The samples were then centrifuged at 512.2× *g* for 20 min, and syneresis was calculated using Equation (3).

(3) $$Syneresis\;\left(\%\right)=\frac{Weight\;of\;supernatant\;\left(g\right)}{Weight\;of\;fermented\;milk\;sample\;\left(g\right)}\times 100$$

#### 2.3.6. Determination of Water-Holding Capacity Throughout Shelf Life

The water-holding capacity (WHC) of the fermented milk samples was determined, in triplicate, using the method described by Harte et al. [17] with slight modifications. Twenty grams of yogurt were weighed, placed into a 50 mL test tube, and centrifuged (Nüve-Bench Top Centrifuge, NF 1200R, Ancara, Turkey) at 15,000× *g* for 15 min at 20 °C. The WHC percentage was calculated using Equation (4).

(4) $$WHC=1-\frac{Serum\;weight\;after\;centrifugation}{Weight\;of\;fermented\;milk\;sample}\times 100$$

#### 2.3.7. LAB Viability Throughout Shelf Life

The counts of *Lc. paracasei* GV17 and the commercial coculture of *S. thermophilus* STI-12 were assessed in triplicate on days 1, 14, and 28. *S. thermophilus* colonies were counted on M17 agar, while *Lc. paracasei* was counted on MRS agar. Both types of plates were incubated at 37 °C under aerobic conditions for 48 h. Viability was expressed as log CFU/mL of fermented milk.

### 2.4. Statistical Analyses

Statistical analysis was conducted with InfoStat version 2020 [18] (InfoStat Software, Córdoba, Argentina). The data were analyzed using one-way analysis of variance (ANOVA) and further examined with the Tukey test (*p* < 0.05).

## 3. Results

### 3.1. Culture Conditions

**Figure 1:**
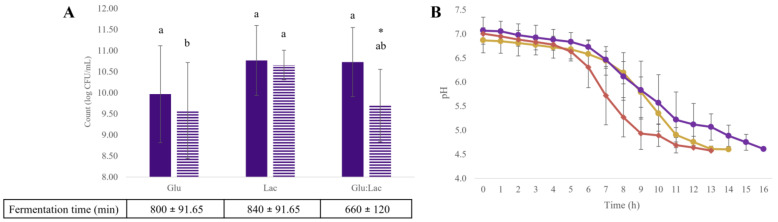
The effects of coculture fermentation on cell counts *Lacticaseibacillus paracasei* GV17 (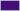) and *Streptococcus thermophilus* STI-12 (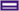) in glucose media (Glu), lactose (Lac), and glucose–lactose (Glu:Lac) at pH 4.6 (**A**) and pH during fermentation in glucose media (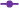), lactose (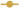), and glucose–lactose (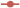) (**B**) Different lowercase letters indicate significant differences (*p* < 0.05) be-tween the media evaluated for the same strain. * For different strains, they indicate significant differences (*p* < 0.05) between strains in the same medium. The results are expressed as mean ± standard deviation (*n* = 9).

When assessing the effect of glucose (Glu), lactose (Lac), and a glucose mixture (Glu:Lac) on cocultures of *Lacticaseibacillus paracasei* GV17 and *Streptococcus thermophilus* STI-12, the pH decline was fastest in the Glu medium, followed by the Lac medium (Figure 1B). In the Glu medium, the combination of GV17 with the commercial strain STI-12 took 180 min longer to reach a pH of 4.6 compared to the Glu:Lac medium (Figure 1A).

In the evaluation of the cocultures, no significant difference (*p* > 0.05) was observed in the growth of GV17 across the three media tested. However, for STI-12, the Lac (10.66 log CFU/mL) medium showing a higher count (*p* < 0.05) was compared to the Glu (9.58 log CFU/mL) medium (Figure 1A). Only in the Glu:Lac medium was there a difference in the counts between the coculture strains, with a count of 10.73 log CFU/mL for GV17 and 9.70 log CFU/mL for STI-12.

**Figure 2:**
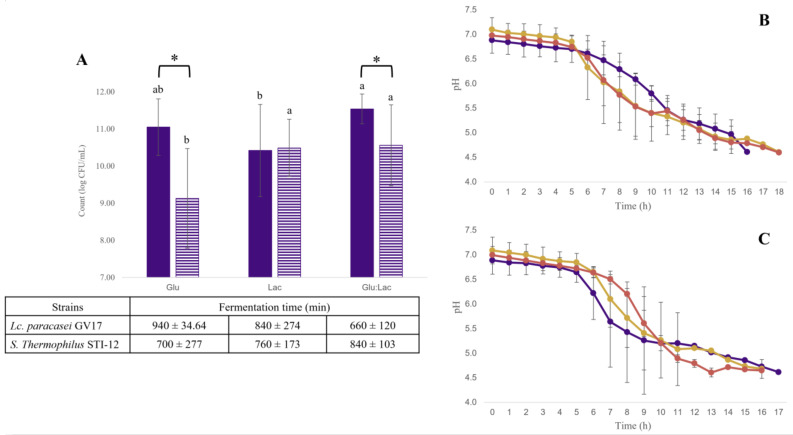
Effects of monoculture fermentation on the cell counts of *Lacticaseibacillus paracasei* GV17 (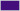) and *Streptococcus thermophilus* STI-12 (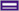) in glucose media (Glu), lactose (Lac), and glucose–lactose (Glu:Lac) at pH 4.6 (**A**) and pH during fermentation in glucose media (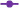), lactose (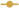), and glucose–lactose (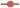) for monocultures of Lacticaseibacillus paracasei GV17 (**B**) and Streptococcus thermophilus STI-12 (**C**). Different lowercase letters indicate significant difference (*p* < 0.05) between the media evaluated for the same strain. * For different strains, they indicate significant difference (*p* < 0.05) between strains in the same medium. The results are expressed as mean ± standard deviation (*n* = 9).

In the separate evaluations of the GV17 and STI-12 monocultures, it was observed that the time to reach a pH of 4.6 was approximately 16 h (Figure 2B,C). Unlike when the strains were added together, the GV17 strain reached a pH of 4.6 more quickly in the Glu medium. In the Lac medium, both monocultures exhibited similar counts (*p* > 0.05); however, in the Glu and Glu:Lac media, the highest counts were observed for *Lc. paracasei* GV17, with 11.05 log CFU/mL and 11.54 log CFU/mL, respectively (Figure 2A).

### 3.2. Estimation of LAB Multiplication Parameters During Fermentation

**Figure 3:**
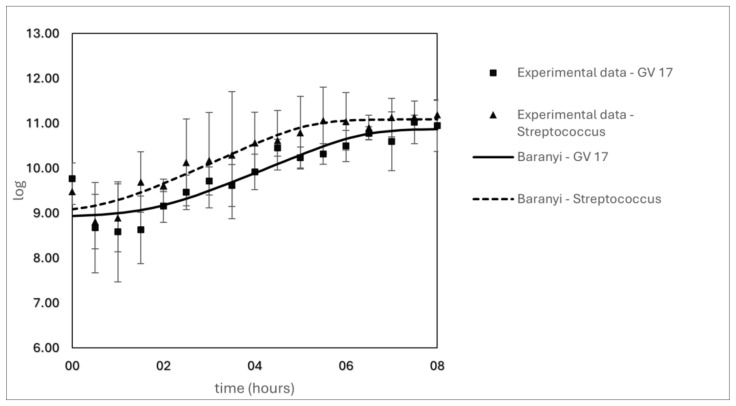
Growth curves of *Lacticaseibacillus paracasei* GV17 and coculture of *Streptococcus thermophillus* STI-12, in fermented milk.

The growth patterns of GV17 and STI-12 are shown in Figure 3. The growth curves of the tested LAB displayed a typical sigmoidal pattern. The initial concentrations were 9.80 log CFU/mL for *Lc. paracasei* GV17 and 9.50 log CFU/mL for *S. thermophilus* STI-12. At the beginning of fermentation, the pH of the fermented milk was 6.37, and the titratable acidity was 0.25 g lactic acid/100 mL.

A pH of 4.6 was reached after eight hours of fermentation. At this time, the concentrations of *Lc. paracasei* GV17 and *S. thermophilus* STI-12 were 10.90 log CFU/mL and 11.20 log CFU/mL, respectively. The titratable acidity at the end of fermentation was 0.72 g lactic acid/100 mL.

In the first hour of fermentation, there was a reduction in the LAB count, with values of 8.59 log CFU/mL for GV17 and 8.90 log CFU/mL for STI-12. This reduction was likely due to the LAB adaptation period during the latency phase. From the second hour to the fifth hour of fermentation, the logarithmic phase was observed, with counts of 10.20 log CFU/mL for GV17 and 10.80 log CFU/mL for STI-12. After 7.5 h, both strains reached the stationary phase with viable bacterial counts greater than 11.00 log CFU/mL (Figure 3).

The fermentation conditions used for evaluating “culture conditions”, such as cocultures (Figure 1B), differed from those applied in the fermented milk production process (Figure 3). In the former, 0.1% of each coculture and 10% milk were added to the medium, whereas in the fermentation process, conditions similar to those used in an industrial setting were applied. These included the addition of 12% total solids, 2% of each coculture (totaling 4%), and 7% sugar to the milk—factors that can contribute to a reduction in fermentation time, as shown in Figure 3.

The maximum specific growth rates (μ_max_) were 0.912 log CFU/h for *Lc. paracasei* GV17 and 0.882 log CFU/h for the co-cultivation of *S. thermophilus* STI-12. The latency times were 2.428 h and 1.943 h for GV17 and STI-12, respectively. The correlation coefficients (R^2^) for the Baranyi model were 0.760 for GV17 and 0.852 for (Table 1).

Some factors may contribute to the slightly lower R^2^ value for GV17, an autochthonous LAB strain from Minas artisanal cheese. Wild LAB strains exhibit greater genetic variability, leading to a more heterogeneous response under certain environmental conditions, and their multiplication is less predictable due to natural adaptations [19]. In contrast, commercial strains, such as STI-12, have optimized growth, resulting in a more linear multiplication pattern that better aligns with mathematical models.

### 3.3. Determination of Syneresis, Water-Holding Capacity, and Viability of LAB Throughout Shelf Life

Table 2 shows the syneresis results over the 28 days of storage of the fermented milk. During the first 14 days, no significant changes were observed (*p* > 0.05). However, after 28 days of storage, there was a reduction of 8.90% in syneresis values (*p* < 0.05).

The values found for water-holding capacity (WHC) explain the results observed in the syneresis analyses, as they showed an inverse relation (Table 2). During the 28 days of shelf life for the fermented milk, there was no statistically significant increase (*p* > 0.05) in WHC.

Throughout the entire storage period of the fermented milk, the viability of GV17 and STI-12 remained above 10.00 log CFU/mL (Table 3). The potentially probiotic LAB strain GV17 showed an increase of approximately 1.00 log CFU/mL (*p* < 0.05) in count after 14 days of refrigerated storage but exhibited no significant difference in count after 28 days. The commercial strain STI-12 did not exhibit a significant increase in viability during the storage of fermented milk at 4 °C.

## 4. Discussion

In food fermentation, reducing fermentation time is a relevant factor, which refers to the duration required for milk to reach a pH of 4.6. Prolonged fermentation can negatively impact large-scale production by extending the production cycle and increasing associated costs [20]. Therefore, shortening the fermentation time can help lower production costs and enhance profitability [2]. Recent studies have demonstrated that the use of exogenous LAB can improve food quality and flavor while simultaneously reducing fermentation time.

During the fermentation process, when the pH of milk reaches approximately 5.5, the multiplication of *S. thermophilus* is typically restricted. Consequently, the β-galactosidase gene (*lacZ*) can remain active under acidic stress, allowing certain *Lactobacillus* species to adapt to this relatively acidic environment and further intensify the acidification process until a pH of 4.6 is reached [21].

Balthazar et al. [22] observed that simultaneously inoculating *S. thermophilus* TH-4^®^ and *Lactiplantibacillus plantarum* B2 in sheep’s milk significantly reduced the fermentation time compared to the addition of *Lp. plantarum* B2 alone. The authors propose a proto-cooperative interaction among the LAB strains tested. *S. thermophilus* produces a well-known cofactor during yogurt fermentation that not only accelerates the growth of LAB but also plays a crucial role in determining the acidification rate of *S. thermophilus* [23].

The symbiotic relationship between *Lactobacillus delbrueckii* subsp. *bulgaricus* and *Streptococcus thermophilus* during their multiplication is well known. When co-cultivated, both strains exhibit enhanced growth, along with an improved acid production rate and yield [20]. In this study, both the indigenous and commercial cultures performed efficiently when combined or added individually to the medium. However, identifying optimal combinations of starter strains is crucial for dairy production, as the interaction between two strains can vary depending on their specific characteristics and metabolic interactions [24].

The viability of LAB in this study was higher than that observed in commercial strains of *Lactobacillus bulgaricus* and *S. thermophilus* used to manufacture lactose-free yogurt with added sugar. At the end of fermentation, values of 7.20 log CFU/mL and 10.20 log CFU/mL were reported, respectively [25].

LAB cells frequently self-regulate their metabolism and initiate stress responses to withstand environmental stressors [2]. Mechanisms of stress response in LAB strains have been identified, including the regulation of cell membrane integrity, fatty acid distribution in the cell membrane, intracellular pH, ammonia content, H-ATPase activity, and the activation of cellular carbohydrate, amino acid, and fatty acid metabolism [26].

Regarding organic acids, the levels and types produced during the fermentation process depend on the LAB strain, culture composition, and multiplication conditions. Some organic acids produced by LAB play a crucial role in the food industry due to their ability to enhance food quality and safety [27]. However, compared to other strains, certain acids require particular attention, such as formic acid, which is one of the most critical symbiotic substances produced by *Streptococcus thermophilus* to promote the growth of certain *Lactobacillus* species.

The acidification curve indicates the rate of lactic acid production, the estimated time to achieve the target pH, and the overall efficiency of the fermentation process [28]. The reduction in pH and the rise in titratable acidity can be attributed to the high metabolic activity of LAB. These bacteria hydrolyze lactose, produce lactic acid, and break down fatty acids and fiber into uronic acids, leading to a decrease in pH values [28].

During the initial phase of fermentation, pH values can drop quickly, which may be associated with the accumulation of organic acids. Additionally, the production of carbon dioxide during fermentation can also contribute to the pH decrease. In a study by Yang et al. [29], the titratable acidity of the samples increased throughout the fermentation process, indicating that the rise in acidity generally aligned with the decline in pH.

Fermented milk, due to lactose hydrolysis and even the addition of sugar, can enable lactic acid bacteria (LAB) to enter their logarithmic phase in approximately two hours. When evaluating the microorganism multiplication model, the logarithmic phase is of particular interest in the production of fermented dairy products, as it is during this phase that substrate fermentation occurs, leading to the production of organic acids and a consequent increase in cell mass over time [30].

The occurrence of a shorter fermentation time may be due to the availability of glucose in lactose-free milk, a result of the β-galactosidase activity added before milk fermentation. Starter cultures such as *S. thermophilus* utilize common hexoses, primarily glucose, which can be metabolized via the Embden–Meyerhof pathway [31].

Lactose and glucose are the main fermentable sugars in milk. LAB converts lactose into lactic acid through homolactic fermentation or heterolactic fermentation [32]. The term lactic acid bacteria (LAB) refers to a group of microorganisms with the metabolic capacity to ferment various carbohydrates, producing primarily lactic acid [33]. One such carbohydrate is the disaccharide lactose, which consists of two simple sugars: glucose and galactose.

Once internalized by the cell, lactose can be metabolized via the Leloir and Tagatose-6-phosphate pathways. In the bacterial cytoplasm, lactose is hydrolyzed by the enzyme beta-galactosidase, which generates the monosaccharides glucose and galactose, making them available for use in the fermentation pathways [34]. In lactose-free milk, this hydrolysis has already occurred. Since glucose is a simpler monosaccharide, its utilization is more rapid, potentially enhancing the metabolism of the added LAB.

Lactose, a disaccharide, needs to be hydrolyzed during the fermentation process by intracellular β-galactosidase, which breaks it down into glucose and galactose [35]. According to Junaid et al. [36], the best fermentation time coefficients are represented by samples made with hydrolyzed milk. This correlation may arise from the pre-existing hydrolysis of lactose, which optimizes the metabolic process in hydrolyzed milk.

Among the technological characteristics of fermented milk, a reduction in syneresis is a desirable characteristic in fermented milk. The lower the syneresis over time, the less unwanted release of the liquid phase from lactose-free fermented milk [37].

Water-holding capacity is a crucial indicator of fermented milk stability [38]. Syneresis is linked to the organization and compaction of fermented milk structure [39]. High syneresis values suggest that water is easily lost from fermented milk, indicating a disorganized structure. In contrast, lower syneresis values imply a more organized structure, particularly in the fats and proteins within fermented milk [40].

The lower degree of syneresis in the fermented milk over the 28 days may be related to a possible increase in the product’s acidity. Even though the fermented milk was stored at low temperatures, the fermentation process could have still continued and further restructured the protein network [41].

Pereira et al. [5] assessed the syneresis of lactose-free milk made with a commercial culture containing *Lactobacillus acidophilus* LA-5, *Bifidobacterium lactis* BB-12, and *Streptococcus thermophilus* (Bio Rich^®^) and supplemented with inulin. The study reported syneresis values of 3.58% on the first day after fermentation and 7.15% after 28 days. The authors suggest that inulin interacts with milk proteins, enhancing the stability of the protein network and forming a protein matrix that is less susceptible to whey expulsion, which accounts for the lower syneresis values.

Tao et al. [42] evaluated the WHC in lactose-free fermented milk and observed a higher value after 14 days of refrigerated storage. The authors attributed this result to the exopolysaccharides produced during lactose hydrolysis. When the exopolysaccharide content is insufficient to fully surround the milk proteins, protein condensation may occur. This can lead to protein binding at multiple surfaces, consequently reducing the stability of the system’s suspension.

There are reports that lactose hydrolysis influences the fermentative properties of yogurt, such as shorter or longer fermentation times, lower viscosity, and higher concentrations of exopolysaccharides [43]. Yamamoto et al. [13] asserts that variations in fermentative properties are strain-dependent, and it is crucial to understand how lactose hydrolysis affects each specific strain.

Yamamoto et al. [13] observed a 1.4-fold increase in the number of viable *Lactobacillus bulgaricus* 2038 cells in lactose-free milk compared to milk containing lactose. Formic acid, a key symbiotic substance produced by *S. thermophilus*, is known to enhance the growth of *Lactobacillus bulgaricus* [44]. The study by Yamamoto et al. [13] found that *S. thermophilus* 1131 generated a higher concentration of formic acid in glucose medium (lactose-free milk) compared to lactose medium during fermentation.

The rise in viable bacterial counts of both strains during storage may be attributed to an adaptive process by LAB, allowing them to utilize the metabolites present in lactose-free hydrolyzed milk. Some studies have reported that storing products from LAB fermentations can sometimes reduce the number of viable cells [45].

At the end of the fermentation process, when a pH of 4.6 was reached, the LAB count was 10.90 log CFU/mL for GV17 and 11.20 log CFU/mL for STI-12 (Figure 3). One day after fermentation, a reduction in the LAB count was observed, with GV17 and STI-12 reaching 10.40 log CFU/mL and 10.72 log CFU/mL (Table 3), respectively. After 28 days of refrigerated storage, the LAB counts for GV17 and STI-12 increased by approximately 0.4 log CFU/mL. This increase in LAB count is not considered sufficient to cause a significant further reduction in pH during storage.

The metabolic activity of LAB fermentation in fermented milk can lead to a gradual decline in pH throughout the product’s shelf life. In a study by Lacerda et al. [46], a decrease of approximately 0.3 in the pH value of fermented milk stored at 4 °C was observed, while a greater reduction occurred when the product was kept at 12 °C. In fermented milk stored at 4 °C, no significant differences in LAB count were detected throughout the shelf life.

## 5. Conclusions

The tested strains of *Lacticaseibacillus paracasei* GV17, in association with the commercial culture *Streptococcus thermophilus* STI-12, exhibited sufficient growth to reach high cell density, enter the stationary phase, and adapt well to the Baranyi model. The results for syneresis and water retention capacity were high, highlighting the need for technological adjustments to minimize defects. The LAB strains maintained high and stable counts throughout the 28-day storage period of the fermented milk, demonstrating good adaptation to the lactose-free matrix. The native strain *Lacticaseibacillus paracasei* GV17, isolated from artisanal cheese from Minas Gerais, exhibited promising characteristics for potential use in functional products.

## Figures and Tables

**Table 1:** Growth parameters of *Lacticaseibacillus paracasei* GV17 and coculture of *Streptococcus thermophilus* STI-12, in lactose-free fermented milk, obtained from the Baranyi model for primary modeling.

Strains		µ_max_ (log CFU/h)	LT (h)	R^2^
*Lc. paracasei*	GV17	0.912 ± 0.541	2.428 ± 1.210	0.760 ± 0.085
*S. thermophilus*	STI-12	0.882 ± 0.641	1.943 ± 0.961	0.852 ± 0.034

µ_max_: maximum specific growth rate; LT: latency phase; R^2^: best fit to data.

**Table 2:** Percentage of syneresis and water-holding capacity recorded for fermented milk inoculated with *Lacticaseibacillus paracasei* GV17 associated with commercial coculture of *Streptococcus thermophillus* STI-12, throughout the shelf life of fermented milk.

Strains		Analysis	1st	14th	28th
*Lc. paracasei* associated with *S. thermophilus*	GV17 and STI-12	Syneresis (%)	56.4 ± 2.79 ^a^	56.7 ± 2.99 ^a^	47.8 ± 1.98 ^b^
		WHC (%)	41.3 ± 4.82 ^a^	41.6 ± 2.57 ^a^	45.0 ± 2.08 ^a^

Different lowercase letters on the same row indicate a significant difference (*p* < 0.05) between the periods analyzed by the Tukey test. The results are expressed as mean ± standard deviation (*n* = 9).

**Table 3:** Viability (log CFU/mL) of fermented milk with LAB *Lacticaseibacillus paracasei* GV17 associated with commercial coculture of *Streptococcus thermophilus* STI-12, throughout the shelf life of fermented milk.

Strains		1st	14th	28th
*Lc. paracasei*	GV17	10.40 ± 0.86 ^b^	11.38 ± 0.56 ^a^	11.30 ± 0.46 ^a^
*S. thermophilus*	STI-12	10.72 ± 0.31 ^a^	10.72 ± 0.90 ^a^	11.18 ± 0.65 ^a^

Different lowercase letters on the same row indicate a significant difference (*p* < 0.05) between the periods analyzed by the Tukey test. The results are expressed as mean ± standard deviation (*n* = 9).

## Data Availability

The original contributions presented in this study are included in the article. Further inquiries can be directed to the corresponding author(s).

## References

[1] Garnier L., Penland M., Thierry A., Maillard M.-B., Jardin J., Coton M., Salas M.L., Coton E., Valence F., Mounier J. (2020). Antifungal activity of fermented dairy ingredients: Identification of antifungal compounds. Int. J. Food Microbiol..

[2] Yang H., Hao L., Jin Y., Huang J., Zhou R., Wu C. (2024). Functional roles and engineering strategies to improve the industrial functionalities of lactic acid bacteria during food fermentation. Biotechnol. Adv..

[3] Sadiq F.A., Yan B., Tian F., Zhao J., Zhang H., Chen W. (2019). Lactic acid bacteria as antifungal and anti-mycotoxigenic agents: A comprehensive review. Compr. Rev. Food Sci. Food Saf..

[4] Cunningham M., Azcarate-Peril M.A., Barnard A., Benoit V., Grimaldi R., Guyonnet D., Holscher H.D., Hunter K., Manurung S., Obis D. (2021). Shaping the future of probiotics and prebiotics. Trends Microbiol..

[5] Pereira J.A., Pinto S.S., Dias C.O., Vieira M.P., Ribeiro D.H., Amboni R.D., Fritzen-Freire C.B. (2020). Potentially symbiotic fermented milk: A preliminary approach using lactose-free milk. LWT—Food Sci. Technol..

[6] Moreira T.C., da Silva Á.T., Fagundes C., Ferreira S.M.R., Cândido L.M.B., Passos M., Krüger C.C.H. (2017). Elaboration of yogurt with reduced level of lactose added of carob (*Ceratonia siliqua* L.). LWT—Food Sci. Technol..

[7] Ryan J., Hutchings S.C., Fang Z., Bandara N., Gamlath S., Ajlouni S., Ranadheera C.S. (2020). Microbial, physico-chemical and sensory characteristics of mango juice-enriched probiotic dairy drinks. Int. J. Dairy Technol..

[8] Gonçalves L.D.d.A., Piccoli R.H., Peres A.d.P., Saúde A.V. (2018). Primary and secondary modeling of Brochothrix thermosphacta growth under different temperature and PH values. Food Sci. Technol..

[9] Baranyi J., Roberts T.A. (1994). A dynamic approach to predicting bacterial growth in food. Int. J. Food Microbiol..

[10] Costa I.M., Miranda T.B.A., Magalhães L.M.M., Fafá S.M., Costa T.J.N., Magalhães M.B., Valente G.L.C., Gomes J.E.G., de Assis D.C.S., Vidal A.M.C. (2024). Minas artisanal cheese as a reservoir of potentially probiotic Lacticaseibacillus paracasei GV17 and Lactococcus lactis GV103 and their functional properties and kinetic mechanisms. J. Food Meas. Charact..

[11] Assis G.B.N., Pereira F.L., Zegarra A.U., Tavares G.C., Leal C.A., Figueiredo H.C.P. (2017). Use of MALDI-TOF Mass Spectrometry for the fast identification of gram-positive fish pathogens. Front. Microbiol..

[12] von Mollendorff J.W., Todorov S.D., Dicks L.M.T. (2007). Factors affecting the adsorption of bacteriocins to *Lactobacillus sakei* and *Enterococcus* sp.. Appl. Biochem. Biotechnol..

[13] Yamamoto E., Watanabe R., Ichimura T., Ishida T., Kimura K. (2021). Effect of lactose hydrolysis on the milk-fermenting properties of *Lactobacillus delbrueckii* ssp. bulgaricus 2038 and *Streptococcus thermophilus* 1131. J. Dairy Sci..

[14] AOAC (2006). Método nº 942.15 e 920. 149. Métodos Oficiais de Análise da AOAC International.

[15] Alvarenga V., Brito L.M., Lacerda I.C.A. (2022). Application of mathematical models to validate emerging processing technologies in food. Curr. Opin. Food Sci..

[16] Kieserling K., Vu T.M., Drusch S., Schalow S. (2019). Impact of pectin-rich orange fibre on gel characteristics and sensory properties in lactic acid fermented yoghurt. Food Hydrocoll..

[17] Harte F., Luedecke L., Swanson B., Barbosa-Cánovas G. (2003). Low-fat set yogurt made from milk subjected to combinations of high hydrostatic pressure and thermal processing. J. Dairy Sci..

[18] Di Rienzo J.A., Casanoves F., Balzarini M.G., Gonzales L., Tablada M., Robledo C.W. (2020). InfoStat Versión 2020.

[19] Abedi E., Hashemi S.M.B. (2020). Lactic acid production—Producing microorganisms and substrates sources-state of art. Heliyon.

[20] Bai M., Yang S., Zhao Q., Wang D., Zhang T., Kwok L.Y., Sun Z. (2024). Fermentation characteristics of *Lactobacillus delbrueckii* subsp. bulgaricus T50 and *Streptococcus thermophilus* S10 complex starter: Enhancing fermentation performance, metabolic interaction and storage stability. LWT—Food Sci. Technol..

[21] Guan Y., Cui Y., Qu X., Li B., Zhang L. (2024). Post-acidification of fermented milk and its molecular regulatory mechanism. Int. J. Food Microbiol..

[22] Balthazar C.F., Teixeira S., Bertolo M.R., Ranadheera C., Raices R.S., Russo P., Spano G., Junior S.B., Cruz A.G., Sant’ana A.S. (2024). Functional benefits of probiotic fermented dairy drink elaborated with sheep milk processed by ohmic heating. Food Biosci..

[23] Mahony J., Bottacini F., van Sinderen D. (2023). Towards the diversification of lactococcal starter and non-starter species in mesophilic dairy culture systems. Microb. Biotechnol..

[24] Ge Y., Yu X., Zhao X., Liu C., Li T., Um S., Zhang L., Chen Z., Zhang Z., Song Z. (2024). Fermentation characteristics and post acidification of yogurt by *Streptococcus thermophilus* CICC 6038 and *Lactobacillus delbrueckii* ssp. bulgaricus CICC 6047 at optimal inoculum ratio. J. Dairy Sci..

[25] Miao M., Li S., Yang S., Yan Q., Xiang Z., Jiang Z. (2024). Engineering the β-galactosidase from *Aspergillus oryzae* for making lactose-free and no-sugar-added yogurt. J. Dairy Sci..

[26] Yang H., Yao S., Zhang M., Wu C. (2021). Heat adaptation induced cross protection against ethanol stress in *Tetragenococcus halophilus*: Physiological characteristics and proteomic analysis. Front. Microbiol..

[27] Özcelik S., Kuley E., Özogul F. (2016). Formation of lactic, acetic, succinic, propionic, formic and butyric acid by lactic acid bacteria. LWT—Food Sci. Tecnol..

[28] Xu X., Cui H., Xu J., Yuan Z., Liu X., Fan X., Li J., Zhu D., Liu H. (2022). Effects of different probiotic fermentations on the quality, soy isoflavone and equol content of soy protein yogurt made from soy whey and soy embryo powder. LWT—Food Sci. Tecnol..

[29] Yang Z., Zhu X., Wen A., Qin L. (2022). Development of probiotics beverage using cereal enzymatic hydrolysate fermented with *Limosilactobacillus reuteri*. Food Sci. Nutr..

[30] Cruz L.H.d.O., Nascimento R.M., Ramos G.L.d.P.A., Gonzalez A.G.M., Domingues J.R. (2024). Development of plant-based yogurt from munguba (*Pachira aquatica*) seeds: Stability and predictive growth of lactic acid cultures. Food Biosci..

[31] Zhao J., Liang Y., Zhang S., Xu Z. (2023). Effect of sugar transporter on galactose utilization in *Streptococcus thermophilus*. Front. Microbiol..

[32] Yang Q., Yao H., Liu S., Mao J. (2022). Interaction and application of molds and yeasts in chinese fermented foods. Front. Microbiol..

[33] Iskandar C.F., Cailliez-Grimal C., Borges F., Revol-Junelles A.-M. (2019). Review of lactose and galactose metabolism in lactic acid bacteria dedicated to expert genomic annotation. Trends Food Sci. Technol..

[34] Machado S.G., Freitas R., Martin J.G.P.M., Lindner J.D. (2022). Letes fermetados. Microbiologia de Alimentos Fermentado.

[35] Dekker P.J.T., Koenders D., Bruins M.J. (2019). Lactose-free dairy products: Market developments, production, nutrition and health benefits. Nutrients.

[36] Junaid M., Inayat S., Gulzar N., Khalique A., Shahzad F., Irshad I., Imran M. (2023). Physical, chemical, microbial, and sensory evaluation and fatty acid profiling of value-added drinking yogurt (laban) under various storage conditions. J. Dairy Sci..

[37] Wilbanks D., Lee M., Rahimi Y., Lucey J. (2023). Comparison of micellar casein isolate and nonfat dry milk for use in the production of high-protein cultured milk products. J. Dairy Sci..

[38] Zeineb J., Olfa O., Slah Z., Touhami K., El Halima H. (2020). Co-fermentation process strongly affects the nutritional, texture, syneresis, fatty acids and aromatic compounds of dromedary UF-yogurt. J. Food Sci. Technol..

[39] Molaee P., Reza F., Mortazavian A., Sarem N., Ali M., Akbar G., Khorshidian N. (2020). Comparative effects of probiotic and paraprobiotic addition on microbiological, biochemical and physical properties of yogurt. Food Res. Int..

[40] Tan C., Tian Y., Tao L., Xie J., Wang M., Zhang F., Yu Z., Sheng J., Zhao C. (2024). Exploring the effect of milk fat on fermented milk flavor based on gas chromatography–ion mobility spectrometry (GC-IMS) and multivariate statistical analysis. Molecules.

[41] Deshwal G.K., Tiwari S., Kumar A., Raman R.K., Kadyan S. (2021). Review on factors affecting and control of post-acidification in yoghurt and related products. Trends Food Sci. Technol..

[42] Tao H., Li S.-Q., Fang M.-J., Cai W.-H., Zhang S., Wang H.-L. (2024). The characterization of a low-calorie and lactose-free brown fermented milk by the hydrolysis of different enzymatic lactose. Foods.

[43] Schmidt C., Mende S., Jaros D., Rohm H. (2015). Fermented milk products: Effects of lactose hydrolysis and fermentation conditions on the rheological properties. Dairy Sci. Technol..

[44] Veringa H.A., Galesloot T.E., Davelaar H. (1968). Symbiosis in yoghurt (II). Isolation and identification of a growth factor for Lactobacillus bulgaricus produced by *Streptococcus thermophilus*. Milk Dairy J..

[45] Li S., Tang S., Ren R., Gong J., Chen Y. (2021). Metabolomic profile of milk fermented with Streptococcus thermophilus cocultured with *Bifidobacterium animalis* ssp. lactis, *Lactiplantibacillus plantarum*, or both during storage. J. Dairy Sci..

[46] Lacerda S., Santos M.C.D., Martins O.A., Pereira J.G. (2022). Microbiological and physicochemical characterization of probiotic fermented milk throughout the shelf life under different storage temperatures. Food Sci. Technol..

